# Supplementary material for: Comparative multiomics analysis of cell physiological state after culture in a basket bioreactor
Source: Sci Rep. 2022 Nov 23;12:20161. doi: 10.1038/s41598-022-24687-4 (PMC9686226; doi:10.1038/s41598-022-24687-4)
Supplement: Supplementary file 1 — Supplementary Information 1. [file 41598_2022_24687_MOESM1_ESM.zip › raw data/Metabolomics raw data/Met_results_dir.html]

Directory Tree


# Directory Tree

../results
  
 ├── 1.MetQuant-QC:代谢物质控结果目录
  
 ├── 2.MetAnnotation:代谢物注释结果目录
  
 │ ├── KEGG
  
 │ ├── HMDB
  
 │ ├── Lipidmaps
  
 │
  
 ├── 3.MetDiffScreening:代谢物定量结果目录  
 │ ├── EG.vs.CG
  
 │
  
 ├── 4.MetDiffAnalysis:差异代谢物分析结果目录
  
 │ ├── EG.vs.CG
  
 │
  
 ├── 5.MetKeggEnrichment:差异代谢物Kegg富集分析结果目录
  
 │ ├── EG.vs.CG

---

tree v1.5.3 © 1996 - 2009 by Steve Baker and Thomas Moore   
HTML output hacked and copyleft © 1998 by Francesc Rocher   
Charsets / OS/2 support © 2001 by Kyosuke Tokoro
